# Supplementary material for: Involvement of Spermidine in the Reduced Lifespan of Caenorhabditis elegans During Vitamin B12 Deficiency
Source: Metabolites. 2019 Sep 19;9(9):192. doi: 10.3390/metabo9090192 (PMC6780408; doi:10.3390/metabo9090192)
Supplement: Supplementary file 1 [file metabolites-09-00192-s001.pdf]

Involvement of spermidine in the reduced lifespan of *Caenorhabditis elegans* during vitamin B_{12} deficiency

Tomohiro Bito [1,*], Naho Okamoto [2], Kenji Otsuka [1], Yukinori Yabuta [1], Jiro Arima [1], Tsuyoshi Kawano [1], and Fumio Watanabe [1]

[1] Department of Agricultural, Life and Environmental Sciences, Faculty of Agriculture, Tottori University, Tottori 680-8553, Japan

[2] The United Graduate School of Agricultural Sciences, Tottori University, Tottori 680-8553, Japan

***Correspondence: bito@tottori-u.ac.jp; Tel.: +81-857-31-5443 (T.B.)

Contents

Figure S1. HPLC analysis of polyamines (putrescine and spermidine) found in worms grown in vitamin B_{12}-supplemented and vitamin B_{12}-deficient conditions.

Figure S2. Amino acid analysis of worms grown in vitamin B_{12}-supplemented and vitamin B_{12}-deficient conditions.

Figure S3. Some properties of *Caenorhabditis elegans* arginase

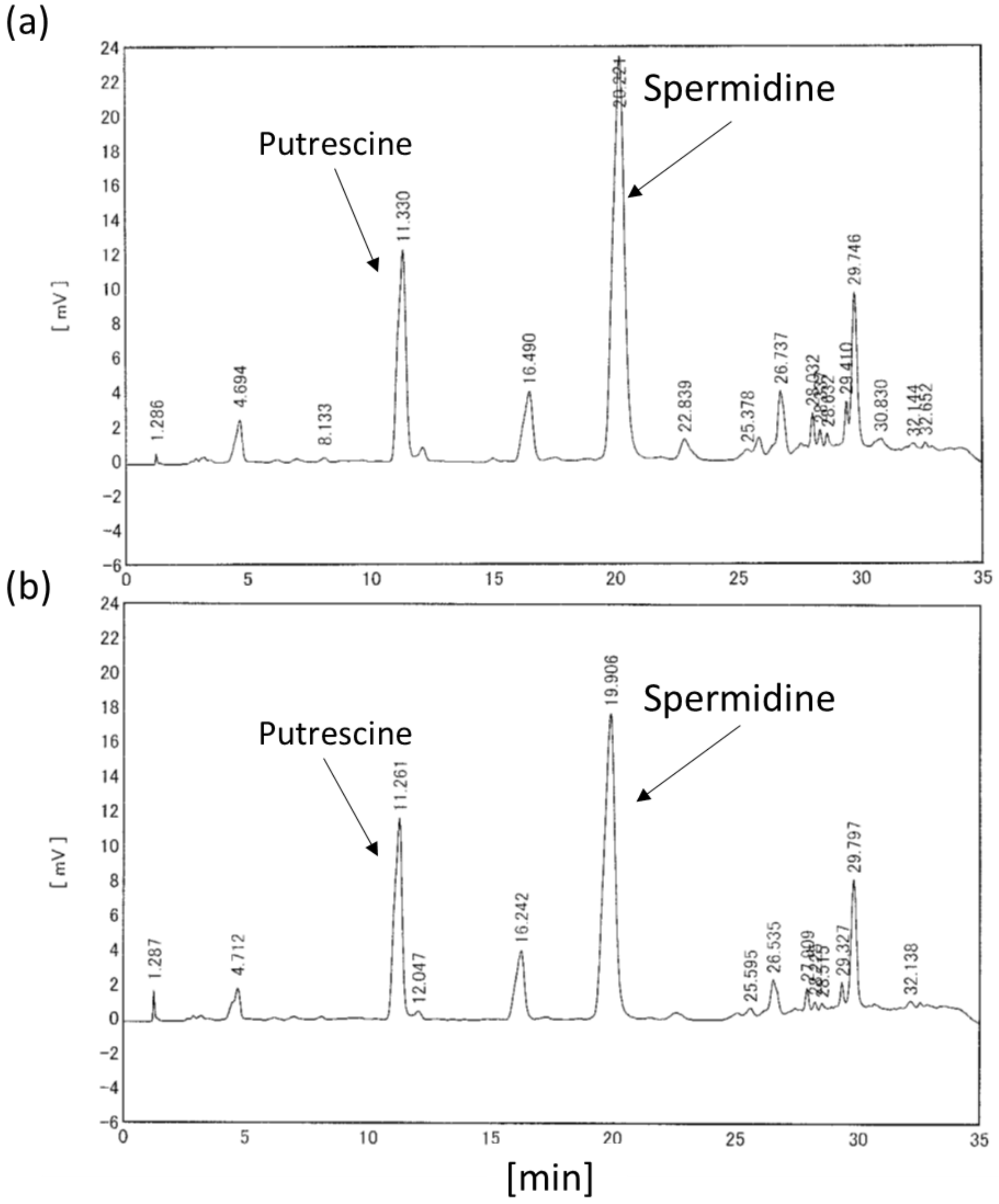

Figure S1. HPLC analysis of polyamines (putrescine and spermidine) found in worms grown under vitamin B_{12}-supplemented and vitamin B_{12}-deficient conditions. Polyamines were analyzed using HPLC as described in Section 2 of the manuscript. Elution patterns of polyamines found in vitamin B_{12}-supplemented worms (control) (a) and in vitamin B_{12}-deficient worms (b). Retention times of putrescine and spermidine were 11.26–11.33 min and 19.91–20.22 min, respectively. Spermine (retention time, approximately 29.0 min) was not detected in *Caenorhabditis elegans*.

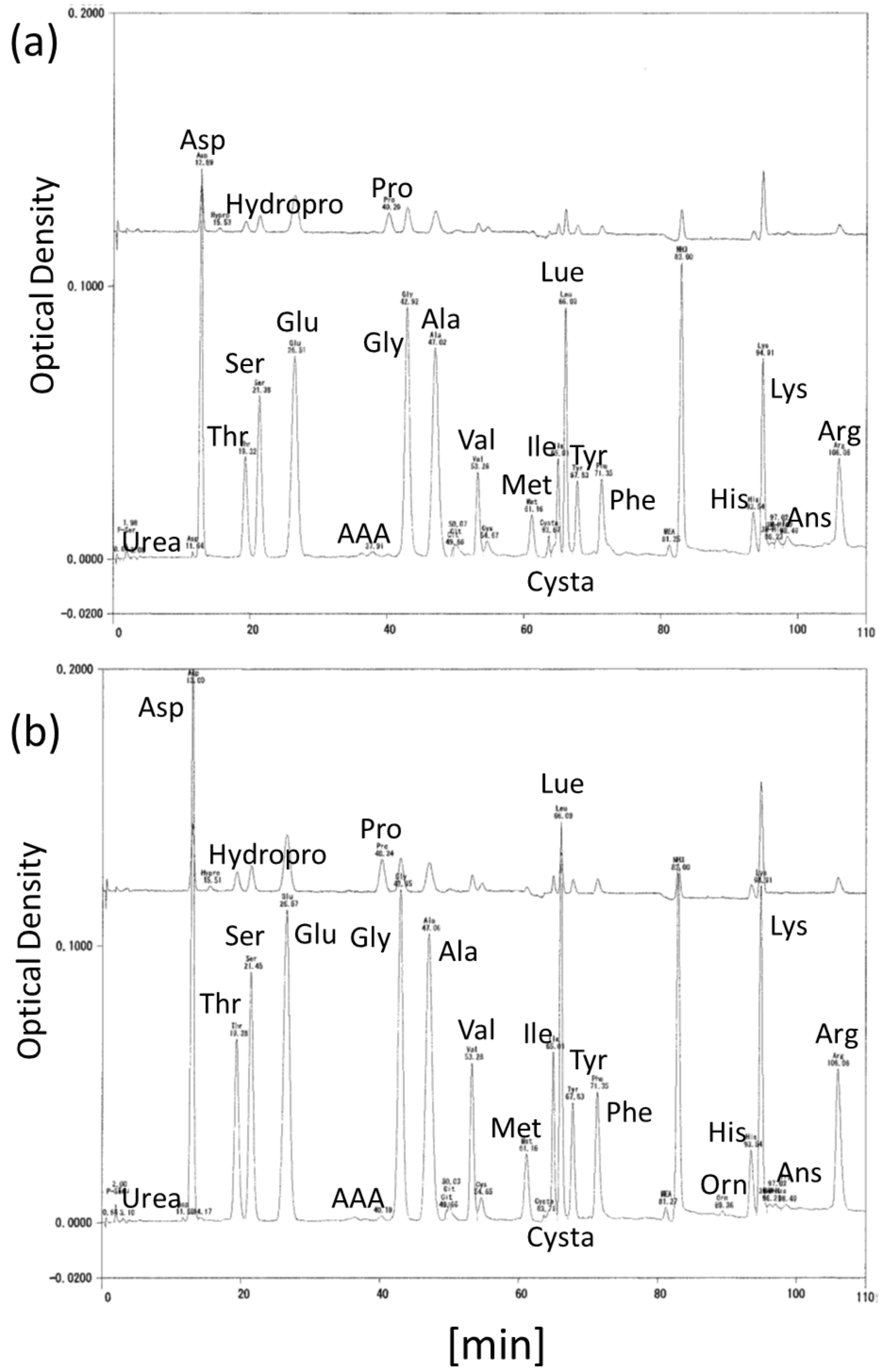

Figure S2. Amino acid analysis of worms grown under vitamin B_{12}-supplemented and vitamin B_{12}-deficient conditions. Elution patterns of amino acids in vitamin B_{12}-supplemented worms (control) (a) and in vitamin B_{12}-deficient worms (b). Amino acid contents of each worm were analyzed using a fully-automated amino acid analyzer (post-column derivatization method). Amino acids were expressed using the three-character notation. AAA and Cysta represent aminoadipic acid and cystathionine, respectively.

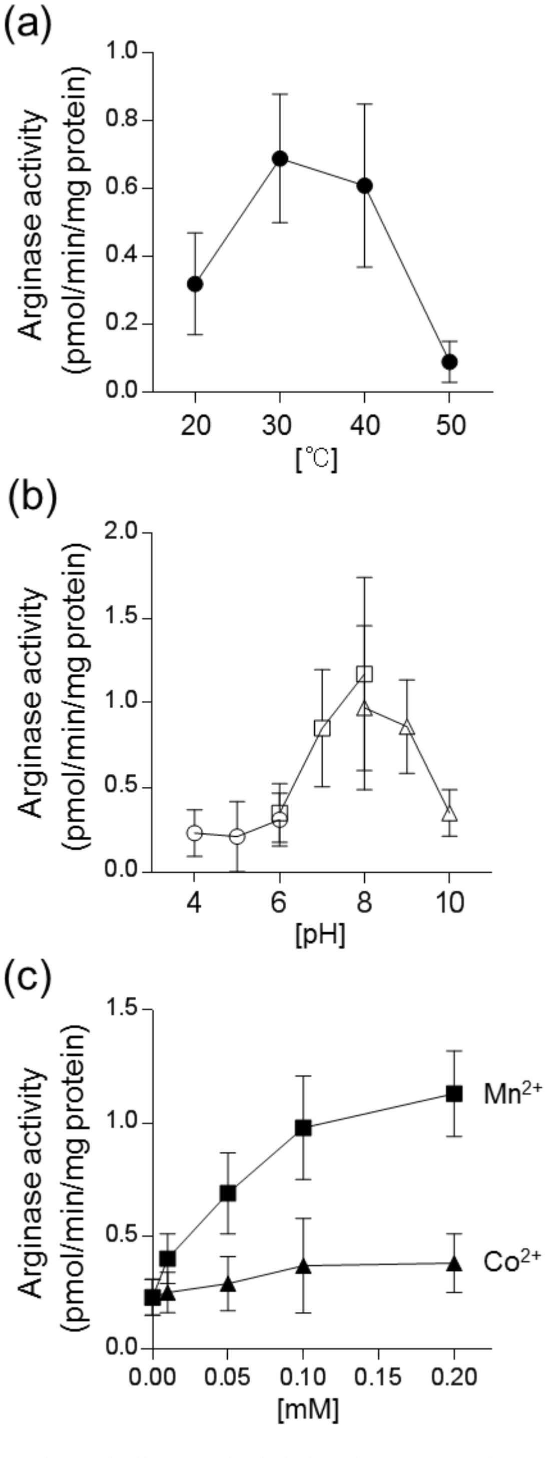

Figure S3. Some properties of *Caenorhabditis elegans* arginase.

Arginase activity was assayed using a commercial arginase assay kit. The crude homogenate of vitamin B_{12}-deficient worms was centrifuged at 15,000 × *g* for 10 min at 4°C, and the supernatant fraction was used as a crude enzyme.

Effect of reaction temperatures on worm arginase activity (a). Arginase activity was assayed at the indicated temperatures. Effect of various pH on worm arginase (b).

Arginase activity was assayed at the indicated pH (acetate-sodium acetate buffer for pH 4, 5, and 6; potassium phosphate buffer for pH 6, 7, and 8; and carbonate-bicarbonate buffer for pH 8, 9, and 10). Effects of various concentrations of Mn (II) and Co (II) on worm arginase activity (c). Arginase activity was assayed in the presence of the indicated amount of $MnSO_4$ or $CoCl_2$.
